# New York Academy of Medicine

**Published:** 1899-03

**Authors:** 


### New York Academy of Medicine.—Section in Orthopaedic Surgery.—Meetings of December 16, 1898, and January 20, 1899.

### SHORTENED PECTORAL MUSCLE.

Dr. R. Whitman presented a patient, a girl 11 years of age, who could not raise her right arm more than 30 degrees above the horizontal. The cause appeared to be obstetrical paralysis. Round shoulders and curvature of the spine were present. He had advised division of the unyielding contraction of the lower border of the pectoralis major muscle, which presented a thick fibrous cord beneath the skin.

Dr. A. B. Judson said that the contraction might have resulted from a paralyzed deltoid which had failed to give normal extension to the pectoral.

Dr. Whitman said that there was a very fair development of the shoulder muscles, and that the curvature of the spine could not be relieved until the contraction that prevented the child from lifting her arm over the head was removed.

### TUBERCULOUS KNEE AND ATHETOSIS.

Dr. Whitman presented a girl 10 years of age who had been under observation for nine years. When one year old and under treatment for disease of the right knee she had a convulsive attack which was followed by right hemiplegia. The return of voluntary power was accompanied by constant convulsive movements of the face, arm and leg, which had continued to the present time and had made treatment of the knee a matter of great difficulty. In spite of splints, traction and plaster of Paris bandages, the convulsive movements of the leg had caused severe pain and prevented repair, so that the local disease was still uncured. But for the youth of the patient, amputation would have been done. The case illustrated the advantages and necessity of rest in the conservative treatment of joint diseases.

CASES OF DOUBTFUL DIAGNOSIS.

Dr. W. R. Townsend presented a boy 11 years of age who fell from a car three months ago and had complained of pain in the left hip ever since. Six weeks ago, when he was first seen, there was symmetry in all the measurements of the lower extremities, but the affected hip showed considerable resistance to motion in any direction, which could sometimes be partly overcome by persuasion and considerable force. Manipulation was painless. He stood and walked with the left foot, leg and thigh everted or rotated outward 90 degrees, and this persisted. By the use of considerable force, the limb could be rolled in, but when released it flew back to its old position. Every muscle reacted perfectly to galvanism and faradism. Tincture of iodine had been used locally, and his locomotion had improved a little. A probable diagnosis of hysteria had been made by exclusion and because he could with effort stand and walk voluntarily in a normal manner and because the bad position could be overcome by a steady pressure and without causing pain.

Dr. Whitman said that a faulty position of a limb in an impressionable patient might be considered as a voluntary or unconsciously selected adaptation to some condition following strain or other injury of a joint.

Dr. Townsend said that the statement had been made that injury of the obturator nerve had in some instances caused a similar eversion, but he had not found any recorded cases.

Dr. G. R. Elliott presented a man 32 years of age. The family history was negative regarding nervous and bony diseases. Five years ago inability to move the left thigh appeared. When motion returned to the left thigh the right was similarly affected. Other symptoms which still persisted were burning sensations in the feet, especially in the heels, great difficulty in standing erect, and walking, and rigidity of the spine preventing him from bending backwards. Tonus palatinus was noted, and there were other degenerative stigmata. The legs were bowed, but otherwise there were no signs of early rhachitic changes. The hamstrings were contracted. There was double hallrix valgus and pes equinus. The upper extremities were normal. There were no sensory disturbances beyond the paræsthesias mentioned. Neurologists had failed to locate any organic nerve lesions. Dr. Elliott was in doubt in regard to the diagnosis. He did not agree with an opinion expressed by some members of the section that it was probably a case of rheumatoid arthritis, a disease which could not present so much disability with practically no involvement of the small joints, almost painless from the beginning and with no deposits about the joints. The pain that was present and the disability were due to the various contractions and consequent disuse of the part's implicated.

CICATRICIAL CONTRACTION OF THE HAND.

Dr. S. Lloyd presented a little boy with cicatricial deformity of the right hand, the result of burns received a year ago. About six weeks ago the little finger, being very much twisted and distorted, was amputated, and superficial tissue was removed from the remaining digits. To replace the cicatricial tissue with normal skin a flap including a little of the fatty tissue was partially dissected from the abdomen, being attached at the top and bottom. Under this the boy's hand was slipped, and a plaster of Paris bandage was applied. This being removed, the attachment of the fingers to the abdomen was very well shown. At a later stage the flap would be entirely detached from the abdomen. There had been no suppuration.

SECONDARY PULMONARY OSTEO-ARTHROPATHY IN A CHILD.

Dr. R. Whitman presented a girl 8 years of age, rather undersized, but in fair physical condition. There was moderate kyphosis and rigidity of the spine, the result of Pott's disease of the tenth dorsal vertebra, accompanied by an abscess in the left iliac fossa, for which she had been treated by the application of a plaster of Paris jacket in 1893, when she was two years old. The abscess disappeared and the patient was recovering favorably till 1896, when persistent cough and expectoration followed an attack of whooping cough. In 1897 enlargement of the fingers was noted, the gait was feeble and shuffling, and there was pain in the knees and ankles with exaggerated patellar reflex and ankle clonus and marked effusion into the knee and ankle joints. The terminal phalanges and the nails were enlarged, and there was cough with abundant expectoration and rales at the apex of the left lung. In 1898 the pain was relieved by the anti-rheumatic administration of salicylate of soda, and although there was marked general improvement the swelling of the knees and ankles persisted, and the increased clubbing of the nails had attracted much attention and was thought to be an instance of the so-called Hippocratic fingers, due to obstruction of the circulation caused by disease of the lungs. Expectoration was moderate in amount, and bacilli were not found. In October, however, an examination showed thickening and enlargement of the bones of the lower arms and sensitiveness to pressure and swelling of the wrist joints. This made the diagnosis clear, and at once connected the clubbing of the fingers, the arthritis and the enlarged bones as symptomatic of the affection known as secondary pulmonary hypertrophic osteo-arthropathy. The child was found to have no psoas contraction or other trace of abscess, and there was apparent recovery from the disease of the spine. There was slight dullness at the apex of the left lung, and increased respiratory sounds at the base of the right. The most marked peculiarity was the great size of the hands as compared with the size of the child, and of the lower arms and legs as compared with the upper segments of the extremities, giving the impression of atrophy of the thighs and upper arms. The bones of the legs and fore-arms were sensitive to pressure. The knees, ankles and wrists were enlarged by an effusion into the joints and by thickening of the surrounding parts without redness, heat or muscular spasm. Motion was very slightly limited. The digits were thickened and their terminal phalanges remarkably enlarged, with nails rose red in color but not especially thickened or curved. The circumference of the ends of the fingers and the breadth of the nails were about twice as great as normal. This condition was somewhat less marked in the feet than in the hands. The affection of the bones in this disease appeared to be a form of malacia in which the organic material is somewhat increased and the mineral substance correspondingly diminished so that the structure of the bone is weakened. The characteristic change in a deposit of new bone beneath the periosteum of the shafts of the phalanges, the metacarpal and metatarsal bones and the lower part of the bones of the lower arm and leg with local sensitiveness, sympathetic arthritis and clubbing of the ends of the digits and hypertrophy of the nails. The affection had been first described in 1888 by Bamburger, and independently by Marie, who differentiated it from acromegalia, with which it had been confounded. In practically all of the cases reported, upward of eighty in number, it was secondary to chronic disease of other parts, in 75 per cent. to tubercular or suppurative disease of the lung or its coverings. The cause of the periosteal and other changes was supposed to be the absorption of irritating substances from the focus of suppuration in or about the lung, combined with impaired circulation. Thus the first evidences appeared in the ends of the fingers. It was a rare disease, and this was believed to be the first typical case reported in a child.

Dr. H. E. Pearse, referring to the great increase in the size of the bones, called attention to the fact that the radiographs showed that the enlargement was longitudinal as well as transverse.

Dr. A. M. Phelps said that he had been impressed with the remarkable bony enlargement. A post mortem examination of the brain and cord would be of great interest. Acromegaly was due to a tumor or growth in one of the ventricles of the brain, and he questioned whether or not in the case presented there was a central lesion due to poisoning from the diseased area. The lungs had not been sufficiently involved to cause obstructed pulmonary circulation. In tabetic joints there was destruction of bone from a central lesion, and cases of rheumatoid arthritis might perhaps have a similar origin and not have been rheumatism at all. He doubted whether such a thing as a simple rheumatic joint existed. They were always multiple.

Dr. R. H. Sayre said that the pathological views presented were not entirely convincing. It was not clear why proliferation of the periosteum should visit the phalanges rather than other parts of the skeleton. In a patient affected with a tubercular knee joint the radiograph had shown a very marked proliferation of the periosteum of the lower end of the femur, and there were marked clubbed fingers. The patient had the appearance of a consumptive in whom the destruction of the lungs was far advanced, but her lungs showed no change. In another patient there was the same condition of the fingers, which were more tender some times than at others. Movement greatly aggravated the inflammation, and nothing gave relief but absolute rest.

Dr. H. S. Stokes said that the etiology was far from being established, as might well be in a disease that had been recognized for only eight or ten years. It had not yet been positively determined even that the condition was dependent on disease of the lungs. If it were, why did it not occur more frequently? In the absence of the characteristic bacilli, it was not certain that the child presented had tuberculosis. In view of her history, it would not have been strange if her general condition had been worse. It was almost impossible to make a diagnosis of lung affections in children with deformed chests. He had seen a specimen of kyphosis from a case in which the diagnosis of tuberculosis of both lungs had been made, and yet at the autopsy the lungs had been found to be normal. Similar cases were not uncommon.

Dr. G. R. Elliott said that if speculation were in order he would agree with Dr. Phelps that the cause of this rare condition was to be sought for through the central nervous system. There was reason to believe that the cause of various distal bony changes and peculiar vascular phenomena presented by the distal extremities, including great sensitiveness, together with certain well marked types of so-called osteo-arthropathy were traceable to central lesions. In the patient presented there were clinical and X-ray evidences of a disturbance of the normal equilibrium between the bone producing and organic producing cells leading to the enlargement. The signs were bi-lateral and symmetrical evidences of central irritation. To say that such a condition was associated with a chronic disease meant very little; to say that it was circulatory was untenable. He believed that the explanation would be found in this—that the trophic and vaso-motor cells had been thrown off the track by some poison, be it tubercular or other, circulating through the central nervous system, selective in its nature and degenerative in its final expression.

Dr. Stokes said that in the five or six autopsies which had been made no nerve lesions had been found, in spite of careful and thorough examination.

Dr. Elliott said that that was true of other diseases which were considered to be due to central nervous lesion.

Dr. Whitman said that many cases of osteo-arthropathy were probably not true examples of the disease in question. In many the only change observed was clubbing of the fingers, which was sometimes seen in cases of simple obstruction of the circulation, described by Hippocrates as a symptom of advanced phthisis and not very uncommon in cases of empyema of long standing. One fact had been established, viz.: that hypertrophic osteo-arthropathy was practically always secondary to some chronic disease, in the case presented, for example, to Pott's disease and chronic bronchitis.

Speculation as to its cause would seem to be less important than further and more careful descriptions and classification of cases.

### CYST OF FEMUR, DOUBLE COXA VARA.

Dr. Whitman presented a boy 11 years of age, with evidences of femur rhachitis and the usual signs of double coxa vara. For several years he had complained of discomfort and pain in the left hip and thigh, the pain at times being severe, especially after exertion. When about five years of age he was treated by a physician for eighteen months for supposed hip disease, and a year later by the application of a plaster jacket for spinal deformity. On September 8, 1898, an operation was begun for the correction of the deformity of the femora by removing wedges of bone from the trochanters. On removing the periosteum from the upper end of the left femur, a peculiar dark color and a somewhat reticulated appearance of the bone were noticed, and at the first touch the chisel broke through the brittle cortex and entered a cavity from which spurted a quantity of serum of the color of prune juice. The cavity was of the size of a hen's egg, its base being shut off from the medullary cavity of the diaphysis by a cone shaped projection covered apparently with cartilage. Its upper extremity reached about half way to the apex of the trochanter. Its walls were lined by a smooth fibrous covering which bled profusely on manipulation. As it was feared that the inner part of the femur was weakened by the cyst, and as it was evident that union in case of fracture would be doubtful, to restrain hemorrhage the cavity was simply packed with gauze, which was removed at the end of four weeks, and the boy began to walk about. The sinus closed one month later. It was evident that spontaneous fracture, as in other cases of coxa vara, could not have been long delayed. If the symptoms should recur, a second operation for the removal of the walls of the cyst would be indicated. Cysts of the femur were usually found at the extremity of the diaphysis, most often at its upper extremity. A diagnosis before operation had not been recorded. They were said to be the result of softening or transformation of an originally more solid growth of a cartilaginous or fibro-cartilaginous nature, probably a displaced fragment of epiphyseal cartilage.

Dr. Sayre had examined the boy two or three months ago. As he had not offered to operate, the patient passed out of his care. At that time he had taken a radiograph of the hip and had observed a spot on the femur which might have been the cyst.

VALUE OF RADIOGRAPHS.

Dr. T. H. Meyers said that he had tried, but usually in vain, to detect abscess, tubercular foci, and other lesions in the bones, by means of skiagraphy. In a case of abscess of the head of the tibia an area of diminished density at the site of the abscess had been clearly revealed, with increased density about it, similar to the contrast seen between the center and the periphery of a long bone in any skiagraph.

Dr. Phelps said that a radiograph would usually show a shadow where there was a lesion, but it could not tell what it was. He had been deceived by pictures taken by good machines and had cut down upon lesions which did not exist. It was not possible to diagnosticate lesions of the soft parts by means of radiography, but if an abscess was known to exist it would aid in locating it.

Dr. H. R. Taylor said that radiographs could not, until further improved, be expected to more than indicate certain physical changes in bone. If the structure had become so attenuated by disease that the X-ray could pass, the focus of disease would be indicated, not otherwise. Intelligence and experience should be brought to the interpretation of these pictures, which are subject to all the distortions of shadows and the errors of photographic processes. A radiogram which was said to reveal the epiphyseal line had really shown a crack in the photographic film. He had a picture of tuberculosis of the carpus in which the diseased foci were shown with the greatest clearness. A cyst of the bone would be revealed if the walls were sufficiently thin to allow the rays to pass.

Dr. Whitman thought that all X-ray pictures should be interpreted. They were of great service to one who had clear ideas of what he was looking for.

POTT'S DISEASE—DEATH CAUSED BY AN ABSCESS IN THE THORAX.

Dr. Whitman also related the history of a boy of 4 years of age who, with an angular projection at the fourth dorsal vertebra, was subject to occasional prolonged asthmatic attacks of such severity that fatal asphyxiation seemed to be imminent. The character of the dyspnœa seemed to warrant a diagnosis of abscess pressing upon the trachea. A plaster jacket and jury-mast were applied with good effect, and a month later the jacket was removed for the purpose of examining the chest more carefully, but the symptoms of dyspnœa, caused, apparently, in part by the removal of the support and in part by the recumbent position, became so urgent that it was immediately reapplied without further examination. The boy died suddenly that evening. The internal organs showed no sign of disease. On removing the lungs and heart, a tense fluctuating tumor was apparent in the median line, the size of a large hen's egg, between the œsophagus and the anterior longitudinal ligament, on a level with the upper border of the third dorsal vertebra, its apex at the sixth dorsal. The abscess contained about two ounces of purulent fluid. It appeared to have escaped from behind the longitudinal ligament into the retro-œsophageal space at about the time of the greater obstruction of breathing, or about six weeks before death. The greatest projection of the tumor was opposite the third vertebra, where it was forced forward, by the spine above the collapsed vertebral body, against the trachea near its bifurcation. An abscess obstructing the respiratory passages in the upper cervical region could be reached and evacuated, but within the chest walls its diagnosis and treatment were not easy. The significance of what might be called asthmatic breathing as distinguished from the embarrassed respiration symptomatic of Pott's disease in this region, should be borne in mind. If the abscess were large and could be percussed posteriorly, costo-transversectomy would be indicated. But in this case there was no dullness on percussion, and the small abscess lay at a distance of three inches from the exterior of the body, so that it was probable that the large opening of so-called posterior thoractomy would mave been necessary, a justifiable operation if the difficulties of diagnosis could have been overcome.

Dr. Phelps recalled two cases in which the abscess had ruptured into the lung, but in neither did suffocation, which had caused death in Dr. Whitman's patient, occur. On the other hand, he had seen cases of cervical diseases in which the abscesses had ruptured into the pharynx and caused suffocation. When the abscess was high enough, it should be opened the very moment it was detected, by an external incision, for it might rupture during sleep at any time, and, if the patient did not suffocate, he would die later of tuberculosis due to infection of the lung.

Dr. A. B. Judson said that the walls of the trachea were not easily compressible except by force, as by the grasp of a strangler or the hangman's rope. When a foreign body in the gullet produced suffocation it was from spasm of the glottis, and not from compression of the trachea. At the level of the third dorsal vertebra, however, the trachea occupied, together with the œsophagus and the deep cardiac plexus of the sympathetic nerve, a narrow strait bounded behind by the vertebral bodies and in front by the upper piece of the sternum, and here, if at any point, its lumen might be diminished by the pressure of a fluctuating tumor. Above this level and below, where the anterior and posterior walls of the thorax diverged, no such pressure was likely to occur. It was not uncommon for abscesses, as in Dr. Whitman's patient, to occupy this critical position. The conservative tendency of cold abscesses to move where there was least resistance often perhaps prevented interference with the vital function of the trachea. He suggested that the fatal result might have been due to spasm of the glottis following the passage of a part of the contents of the abscess into the trachea, or to some interference with the cardiac plexus.

Dr. Taylor was reminded of a case of Pott's disease reported by Dr. W. R. Townsend, in which the abscess was in this region. An unusual form of dyspnœa was a feature of the clinical history, and the child died suddenly a few days after admission to the hospital. A rather small abscess which had not ruptured was found in front of the spinal column at the root of the neck. It was supposed that suffocation had been due to some traction upon the nerves rather than to pressure.

Dr. Myers recalled, and continued, the history of a boy, 7 years of age, who was before the section on March 18, 1898. (See the TEXAS MEDICAL JOURNAL, August, 1898, p. 70.—EDITOR.) The abscess had burrowed forward into the neck from the fifth dorsal vertebra and discharged behind the right sterno-mastoid. The evening temperature rose two degrees when the boy was allowed to be up and was normal when he was kept recumbent, in which position the drainage was free. He had therefore been kept in bed for two months, after which his general health was entirely restored and the sinus remained closed for several months. He had, however, returned with a profuse recurrence of the discharge, an enlargement of the post-cervical glands on the right side, and an abscess over the manubrium, but with no rise of temperature. An irrigating fluid passed from the old sinus out of the pharynx by a passage which was open for a month but which had been closed for four weeks. It was a question whether one of the abscesses perforated or whether one of the cervical glands ruptured and discharged.

Dr. Judson recalled a case in which, during the progress of purulent hip disease, an abscess over the manubrium turned out to be from caries at the junction of the upper and middle piece of the sternum. There was spontaneous rupture externally, consolidation, a scar attached to the bone, and recovery with angular deformity, anterior instead of posterior as in Pott's disease. The angle formed by the manubrium and the gladiolus measured more than twenty-five degrees. The sinus had closed seventeen years ago. Recovery from the hip disease had been very favorable, and the caries of the sternum had left no inconvenience.

Dr. Homer Gibney said that it was reasonable to believe that abscesses occurred as often with disease of the upper dorsal region as of the cervical, but they were not so easily detected in the former and were too often overlooked.

Dr. Whitman said that in the case reported by him the abscess had not ruptured, it was strictly confined to the retro-œsophageal space in front of the spine. There had been no change in voice or difficulty in swallowing. An abscess in this region was a direct menace to life, the dangerous symptom being attacks of inspiratory dyspnœa. It is probable that an operation would have saved life in this case.

ALLUMINUM CORSET.

Dr. Phelps exhibited an alluminum corset for the treatment of spinal disease. He had experimented largely with various materials, such as sole leather, celluloid, wood, etc., and considered this material, which was light, clean, able to keep its shape and durable, as the best that he had found for the purpose.

